# Genetic diversity and grouping of pigeonpea [*Cajanus cajan* Millspaugh] Germplasm using SNP markers and agronomic traits

**DOI:** 10.1371/journal.pone.0275060

**Published:** 2022-11-03

**Authors:** Esnart Nyirenda Yohane, Hussein Shimelis, Mark Laing, Admire Shayanowako

**Affiliations:** 1 African Centre for Crop Improvement, School of Agricultural, Earth and Environmental Sciences, University of KwaZulu-Natal, Scottsville, Pietermaritzburg, South Africa; 2 Department of Agricultural Research Service, Chitedze Agricultural Research Station, Lilongwe, Malawi; National Cheng Kung University, TAIWAN

## Abstract

Knowledge of genetic interrelationships and grouping among pigeonpea germplasm collections is fundamental to selecting breeding parents with unique genetic constitutions. The objectives of this study were to assess the genetic diversity and genetic grouping present among 81 pigeonpea genotypes collected from Malawi, Tanzania and Kenya using 4122 single nucleotide polymorphism (SNP) markers and complementary morphological traits. The SNP markers and phenotypic traits revealed significant genetic variation among the assessed genotypes. The test genotypes were resolved into three distinct clusters based on both marker systems. The mean gene diversity and the polymorphic information content (PIC) were 0.14 and 0.11, suggesting moderate genetic differentiation among the genotypes. The analysis of molecular variance revealed that differences among populations accounted for only 2.7% of the variation, while within the population (among individuals) accounted for 97.3% of the variation. The results based on the DArT SNP genotyping complemented the phenotypic data and led to the selection of unique pigeonpea genotypes for effective breeding programs in Malawi and related agroecologies. This suggested that unique breeding populations could be created by identifying and selecting divergent individuals as parental lines. There is a need to create a new genetic variation or introgress genes from genetically unrelated parents to increase the genetic base of the current pigeonpea breeding populations.

## Introduction

Pigeonpea is a protein-rich legume crop cultivated in more than 25 tropical and sub-tropical countries either as a sole crop or intercropped with cereals or other legumes. Pigeonpea is also a major income source for many small-scale farmers in Africa and Asia [1]. Pigeonpea has high biomass productivity making it suitable as a fodder crop [2]. Like other legume crops, pigeonpea forms symbiotic associations with nitrogen-fixing bacteria and can potentially fix between 69 to 100 kg ha^-1^ atmospheric nitrogen (N) [3] with a net contribution of 2 to 28 kg N ha^-1^ depending on genotype and environmental factors [4, 5]. Furthermore, its roots help release soil-bound phosphorus to make it available for plant growth [6]. Despite its diverse economic importance, pigeonpea is classified among underutilized and orphaned crop species.

Consequently, the production and productivity of pigeonpea are still low to attract interest from commercial and large-scale farming enterprises. The neglect of orphan crops such as pigeonpea by crop improvement research programs compared to other commodity crops such as maize, wheat, and rice has contributed to a lack of improved and high-yielding cultivars in sub-Saharan Africa (SSA). To date, very few commercially grown pigeonpea varieties are available, meeting farmer and market preferences in SSA. This includes hybrids such as ICPH 2671, ICPH 2740, and ICPH 3762 developed by the International Crops Research Institute for the Semi-Arid Tropics (ICRISAT- India) using the cytoplasmic nuclear male sterility (CMS) system [7]. Nonetheless, the crop has uncharted market potential if the quantity and quality of production are enhanced [2]. Sustainable promotion and advancement of pigeonpea will require developing and deploying improved cultivars acceptable by farmers and the entire value chain.

The development of new cultivars will require understanding the existing diversity to inform breeding programs and germplasm management strategies. Knowledge of the genetic basis of yield, quality and stress tolerance is important for the genetic improvement of pigeonpea. Varshney et al. [8] reported assembled 605.78 Mb of the 833.07 Mb pigeonpea reference genome, which helps in the identification of the genetic basis of agronomically important traits to accelerate the development of improved varieties. The genome analysis predicted 48,680 genes of pigeonpea with potential genes for drought tolerance. Similarly,Singh et al. [9] identified 1,213 disease resistance genes and 152 abiotic stress tolerance genes in pigeonpea, making it a hardy crop. However, there is limited information on the magnitude of genetic diversity within the cultivated pigeonpea gene pool [10]. Knowledge of the genetic basis of yield, quality and stress tolerance is essential for the genetic improvement of pigeonpea. However, there is limited information on the magnitude of genetic diversity within the cultivated pigeonpea gene pool [10]. Morphological traits, biochemical and molecular markers have been used in genetic diversity assessments, genetic grouping and selection programs. The ICRISAT maintains 13,632 pigeonpea accessions, including landraces, cultivars, breeding materials, and wild relatives, which are important genetic resources for maintaining genetic diversity for vital morphological and agronomic traits [11]. Molecular markers are robust compared to morphological and biochemical markers in genetic diversity analysis [12]. Molecular markers offer a viable option to accelerate conventional breeding in pigeonpea or related legumes [13]. Several molecular markers have been used in genetic diversity analysis of pigeonpea, such as the restriction fragment length polymorphism (RFLP) [14], amplified fragment length polymorphism (AFLP) [15], random amplified polymorphic DNA (RAPD) [16], simple sequence repeats (SSR/microsatellites) [17] and single nucleotide polymorphism (SNP) [18]. The SNP markers derived from next-generation sequencing have been widely used because they have greater abundance throughout the genome. The automated data generation and collection make SNPs the preferred markers for all molecular breeding applications [19]. In addition, SNP markers are increasingly time, and cost-efficient to genotype large populations with relatively higher throughput [20]. High density SNP arrays and genotyping by sequencing (GBS) have become attractive genotyping tools in pigeonpea. High–density chip arrays have been developed for pigeonpea for instance 62K genic-SNP chip array for Affymetrix GeneTitan^®^ platform called CcSNPnks’ has been developed [21], which provides an opportunity for the identification of novel QTLs for yield, nutrition quality and resistance to environmental stresses using mapping population and association mapping analysis. Thousands of SNPs detected across the genome are useful for characterizing germplasm and maker-trait association mapping. Yan *et al*. [22] developed a pilot diversity array technology (DArT) library for pigenonpea comprising 5,376 SNPs to analyse 96 genotypes representing 20 *Cajanus* species. The authors reported a narrow range of genetic diversity among the tested genotypes. More than 15000 SNPs were discovered recently across the pigeonpea genome [23].

The recently compiled diversity arrays technology (DArT) library on pigeonpea genome provides opportunities for gene discovery and developing strategies for marker-assisted selection to accelerate breeding progress in pigeonpea. Pigeonpea breeding in Malawi is lagging and is mainly focused on conventional breeding methods. Conventional breeding should be complemented with genomic-assisted selection for precision and accelerated breeding and variety release. Yohane et al. [24] reported significant genetic variation in a diverse panel of pigeonpea. However, it was established that selection was confounded by high environmental variance affecting phenotypic trait expression. Therefore, it was imperative to complement phenotypic data with molecular data to reduce environmental variance and improve genetic grouping and selection efficiency for cultivar development. Therefore, this study aimed to assess the genetic diversity and grouping among 81 pigeonpea genotypes using 4 122 single nucleotide polymorphism markers and complementary morphological traits. The results will assist in parental selection to initiate pigeonpea pre-breeding in Malawi.

## Materials and methods

### Plant materials

A population of 81 pigeonpea genotypes were used for this study. Test genotypes were collected from the Department of Agricultural Research Services, Lilongwe, Malawi (13°59’S 33°38’E, 1146 meter above sea level [masl]) and the National Plant Genetic Resource Centre, Lilongwe, Malawi (13°59’S 33°38’E, 1146 masl), the International Crops Research Institute for the Semi-Arid Tropics, Nairobi, Kenya 1°14’10”S 36°49’07”E 1697 masl), and Tanzania Agricultural Research Institute, Ilonga, Tanzania (6°42”S 37° 2”E 506 masl). The germplasm included landraces, breeding lines, and released cultivars obtained from different sources, as presented in Table 1. The germplasm is important for pigeonpea improvement in Malawi, and the full phenotypic and genotypic characterization of this germplasm is imperative to facilitate its utilization in breeding programs.

**Table 1:** Description of the pigeonpea genotypes used in the study.

Code	Genotype designation/name	Description	Source/origin	Code	Genotype designation/name Description		Source/origin
G1	ICEAP 0673/1	Breeding line	ICRISAT, Kenya	G42	ICEAP 87105	Cultivar	ICRISAT, Kenya
G2	ICEAP 00554	Breeding line	ICRISAT, Kenya	G43	MWPLR 16	Landrace	MPGRC, Malawi
G3	ICEAP 01164/1	Breeding line	ICRISAT, Kenya	G44	TZA 2496	Landrace	TARI, Tanzania
G4	MWPLR 19	Landrace	MPGRC, Malawi	G45	TZA 5582	Landrace	TARI, Tanzania
G5	MWPLR 22	Landrace	MPGRC, Malawi	G46	TZA 5596	Landrace	TARI, Tanzania
G6	ICEAP 01170	Breeding line	ICRISAT, Kenya	G47	Chitedze Pigeonpea 2	Cultivar	DARS, Malawi
G7	ICEAP 01169	Breeding line	ICRISAT, Kenya	G48	MWPLR 7	Landrace	MPGRC, Malawi
G8	TZA 2439	Landrace	TARI, Tanzania	G49	Babati	Landrace	TARI, Tanzania
G9	MWPLR 9	Landrace	MPGRC, Malawi	G50	TZA 5557	Landrace	TARI, Tanzania
G10	MWPLR 6	Landrace	MPGRC, Malawi	G51	MWPLR 14	Landrace	ICRISAT, Kenya
G11	MWPLR 17	Landrace	MPGRC, Malawi	G52	ICEAP 01101/1	Breeding line	ICRISAT, Kenya
G12	TZA 253	Landrace	TARI, Tanzania	G53	TZA 2456	Landrace	TARI, Tanzania
G13	MWPLR 1	Landrace	MPGRC, Malawi	G54	TZA 5464	Landrace	TARI, Tanzania
G14	MWPLR 18	Landrace	MPGRC, Malawi	G55	ICEAP 01101/2	Breeding line	ICRISAT, Kenya
G15	TZA 2464	Landrace	TARI, Tanzania	G56	ICEAP 01285	Breeding line	ICRISAT, Kenya
G16	ICEAP 00604	Breeding line	ICRISAT, Kenya	G57	MWPLR 25	Landrace	MPGRC, Malawi
G17	TZA 2509	Landrace	MPGRC, Malawi	G58	ICEAP 87091	Breeding line	ICRISAT, Kenya
G18	ICEAP 01146/1	Breeding line	ICRISAT, Malawi	G59	TZA 2692	Landrace	TARI, Tanzania
G19	MWPLR 11	Landrace	MPGRC, Malawi	G60	TZA 2807	Landrace	TARI, Tanzania
G20	TZA 5555	Landrace	TARI, Tanzania	G61	ICEAP 00068	Breeding line	ICRISAT, Kenya
G21	No. 40	Landrace	TARI, Tanzania	G62	TZA 2785	Landrace	TARI, Tanzania
G22	ICEAP 01150	Breeding line	ICRISAT, Kenya	G63	MWPLR 10	Landrace	MPGRC, Malawi
G23	MZ2/9	Breeding line	TARI, Tanzania	G64	ICEAP 00612	Breeding line	ICRISAT, Kenya
G24	ICEAP 01172/1	Breeding line	ICRISAT, Kenya	G65	MWPLR 21	Landrace	MPGRC, Malawi
G25	ICEAP 01103/1	Breeding line	ICRISAT, Kenya	G66	TZA 2514	Landrace	TARI, Tanzania
G26	MWPLR 24	Landrace	MPGRC, Malawi	G67	TZA 2466	Landrace	TARI, Tanzania
G27	ICEAP 01155	Breeding line	ICRISAT, Kenya	G68	ICEAP 01179	Breeding line	ICRISAT, Kenya
G28	ICEAP 01180/2	Breeding line	ICRISAT, Kenya	G69	MWPLR 13	Landrace	MPGRC, Malawi
G29	MWPLR 4	Landrace	MPGRC, Malawi	G70	MWPLR 2	Landrace	MPGRC, Malawi
G30	Kachangu	Cultivar	DARS, Malawi	G71	TZA 250	Landrace	DARS, Malawi
G31	Mwayiwathualimi	Cultivar	DARS, Malawi	G72	MWPLR 3	Landrace	MPGRC, Malawi
G32	MWPLR 8	Landrace	ICRISAT, Kenya	G73	TZA 5541	Landrace	TARI, Tanzania
G33	ICEAP 01154/2	Breeding line	ICRISAT, Kenya	G74	MWPLR 23	Landrace	MPGRC, Malawi
G34	Chitedze Pigeonpea 1	Cultivar	DARS, Malawi	G75	ICEAP 00979/1	Breeding line	ICRISAT, Kenya
G35	ICEAP 01164	Breeding line	ICRISAT, Kenya	G76	TZA 197	Landrace	TARI, Tanzania
G36	Bangili	Landrace	TARI, Tanzania	G77	MWPLR 20	Landrace	MPGRC, Malawi
G37	ICEAP 00053	Breeding line	ICRISAT, Kenya	G78	HOMBOLO	Landrace	TARI, Tanzania
G38	MWPLR 12	Landrace	MPGRC, Malawi	G79	ICEAP 86012	Breeding line	ICRISAT, Kenya
G39	TZA5463	Landrace	TARI, Tanzania	G80	ICEAP 01106/1	Breeding line	ICRISAT, Kenya
G40	MWPLR 5	Landrace	MPGRC, Malawi	G81	Sauma	Cultivar	DARS, Malawi
G41	MWPLR 15	Landrace	MPGRC, Malawi				

ICRISAT = International Crops Research Institute for the Semi-Arid Tropics, DARS = Department of Agricultural Research Services, TARI = Tanzania Agricultural Research Institute

MPGRC = Malawi Plant Genetic Resource Centre

### Genotyping

#### DNA extraction and DArT sequencing

Ten seeds of each pigeonpea genotype were planted in plastic pots and allowed to grow for three weeks before DNA was extracted. Fresh leaf samples of 10 individual plants per genotype were pooled so that each genotype was well represented. Similarly, 15 pigeonpea plants per genotype were sampled and bulked DNA used for genetic analysis [25]. This was done to maintain the complex genetic information stored in a highly heterogeneous population considering the level of outcrossing in pigeonpea. The collected leaf samples were stored in a deep freezer at– 80 °C. Deoxyribonucleic acid (DNA) extraction was performed following the Diversity Arrays Technology Sequencing (DArTseq) protocol (https://www.diversityarrays.com/files/DArT). Fifty milligrams of total genomic DNA were extracted from the well-developed trifoliated leaves using the NucleoSpin Plant II kit (Macherrey-Nagel, Duren, Germany) with the Lysis Buffer I (based on the cetyl trimethylammonium bromide (CTAB) method). The DNA quality and quantity of each sample were determined on 2% agarose gel followed by quantification using a Nanodrop 2000 Spectrophotometer (ND-2000 v3.5 NanoDrop, Technologies, Inc). The DNA samples were sent to the Biosciences eastern and central Africa International Livestock Research Institution (BecA-ILRI-hub in Kenya (https://hub.africabiosciences.org/) for genotyping.

### Phenotyping

The phenotypic evaluation of the accessions was conducted in the 2017/18 and 2018/19 growing seasons at Bvumbwe, Chitedze, and Makoka. The Bvumbwe site (15°55′ S 35°04′ E) receives an average of 1208.6 mm of rainfall per year, and the average temperature ranges between 16.2 and 24.9 °C. The Chitedze site (13°59′ S 33°38′ E) receives 811.6 mm of rainfall annually with an average temperature between 18.5 and 29.4 °C. At Makoka (15°32′ S 35°11′ E), the average rainfall received per year is 875.7 mm while the average temperature ranges between 15.6 and 28.2 °C. The full description of the sites’ environmental and edaphic conditions is presented in Yohane et al. [24].

Treatments were laid out using a 9 × 9 alpha-lattice design at each testing location. Each genotype was planted on a plot consisting of two rows. Each row was 5m in length spaced at 0.90 m apart, giving a plot size of 4.5 m^2^. Seeds were planted 0.75 m apart within a row. Three seeds were planted per planting station and thinned to one plant two weeks after emergence. The phenotypic data collected included qualitative and quantitative attributes (S1 Table) following the International Board for Plant Genetic Resource [26] as described in Yohane et al [24].

### Phenotypic data analysis

The analysis of variance (ANOVA) of phenotypic data was presented in Yohane *et al*. [24]. Summary statistics of the phenotypic data were derived in SPSS version 26 [27]. Phenotypic clusters based on the dissimilarity matrix were generated using the Gower method implemented in the “cluster” and “graphics” procedures in R statistical package version 2.1.0. The final hierarchical cluster was constructed using the ward D2 method in “cluster” in R package version 2.1.0. [28].

### Genotyping data analysis

DArTseq SNP delivered markers were filtered for quality control to remove bad SNPs and genotypes using the “impute” package in R software version 1.42.0 [29]. A total of 12,366 SNP markers were identified from the raw data, and after filtering markers and genotypes with > 20% missing data, 20% of heterozygosity, and the MAF value of less than 0.05 were removed, resulting in 4122 informative SNP markers and 81 genotypes that were used for analysis.

The gene diversity, minor allele frequency (MAF), polymorphic information content (PIC), and heterozygosity (Ho) were calculated using the “diveRsity” procedure in R software [30]. The analysis of molecular variance (AMOVA) was conducted using the GenAlex version 6.5 [31].

#### Genetic diversity and grouping

The genetic groups of the 81 genotypes was determined using the admixture model-based clustering method in STRUCTURE Harvester [32]. The burn-in period and Markov Chain Monte Carlo (MCMC) iterations were set at 10,000 to derive the population structure based on 4124 SNP markers distributed across the pigeonpea genome. The K-value was set between 1 and 10 to generate the number of subpopulations in the genotypes. The best K-value with the highest likelihood for estimating a suitable population size for the data set was determined using the Evanno procedure [33]. The accessions with a membership probability ≤ 0.70 of a sub-population were assigned to an admixture group, and those ≥ 0.70 were assigned to a distinct population. The dendrograms were generated using the genetic dissimilarity matrix using the “phylogenetics” and “evolution” procedures in R [34].

### Joint analysis of phenotypic and SNP data

Genetic groups were defined using a combination of the phenotypic and genotypic dissimilarity matrices. The joint matrix was generated by the summation of the genotypic and phenotypic dissimilarity matrices. The phenotypic dissimilarity matrix was generated using Gower’s distance matrix, while the genotypic dissimilarity matrix was based on Jaccard’s coefficients. The groups generated from the phenotypic and genotypic sets were compared using the “viridis” procedure in R version 5.0 [35], and the similarity of the two dendrograms was assessed using tanglegram function developed by the “dendextend” R package version 1.0.1 [36].

## Results

### Genetic diversity and grouping based on SNP markers

#### Genetic diversity parameters

The SNP call rate and average reproducibility ranged from 0.34 to 0.98, and 0.9 to 0.99, respectively. Heterozygosity values varied from 0.21 to 0.23, with a mean of 0.22 (Table 2). Gene diversity ranged from 0.00 to 0.50, with a mean of 0.14. The SNP markers were moderately polymorphic, with PIC values ranging from 0.00 to 0.38 with a mean value of 0.11. The markers included the rare variants with a minimum MAF of 0.00 and common variants with a maximum MAF of 0.50 and a mean of 0.12. The inbreeding coefficient averaged -0.56, showing a high level of heterozygosity. The chromosomes 11 and 2 had the largest density of SNPs among the 11 linkage groups (S1 Fig).

**Table 2:** Diversity parameters of 81 pigeonpea genotypes based on 4122 SNP markers.

Parameter	AvgRep	Call rate	GD	PIC	MAF	Ho	F
**Minimum**	0.90	0.34	0.00	0.00	0.00	0.21	-0.65
**Maximum**	0.98	0.98	0.50	0.38	0.50	0.23	-0.49
**Mean**	0.74	0.74	0.14	0.11	0.12	0.22	-0.56

AvgRep = Average Reproducibility, GD = genetic diversity, PIC = polymorphic information content, MAF = minor allele frequency, Ho = observed heterozygosity, F = inbreeding coefficient

#### Genetic relationships

**Fig 1:**
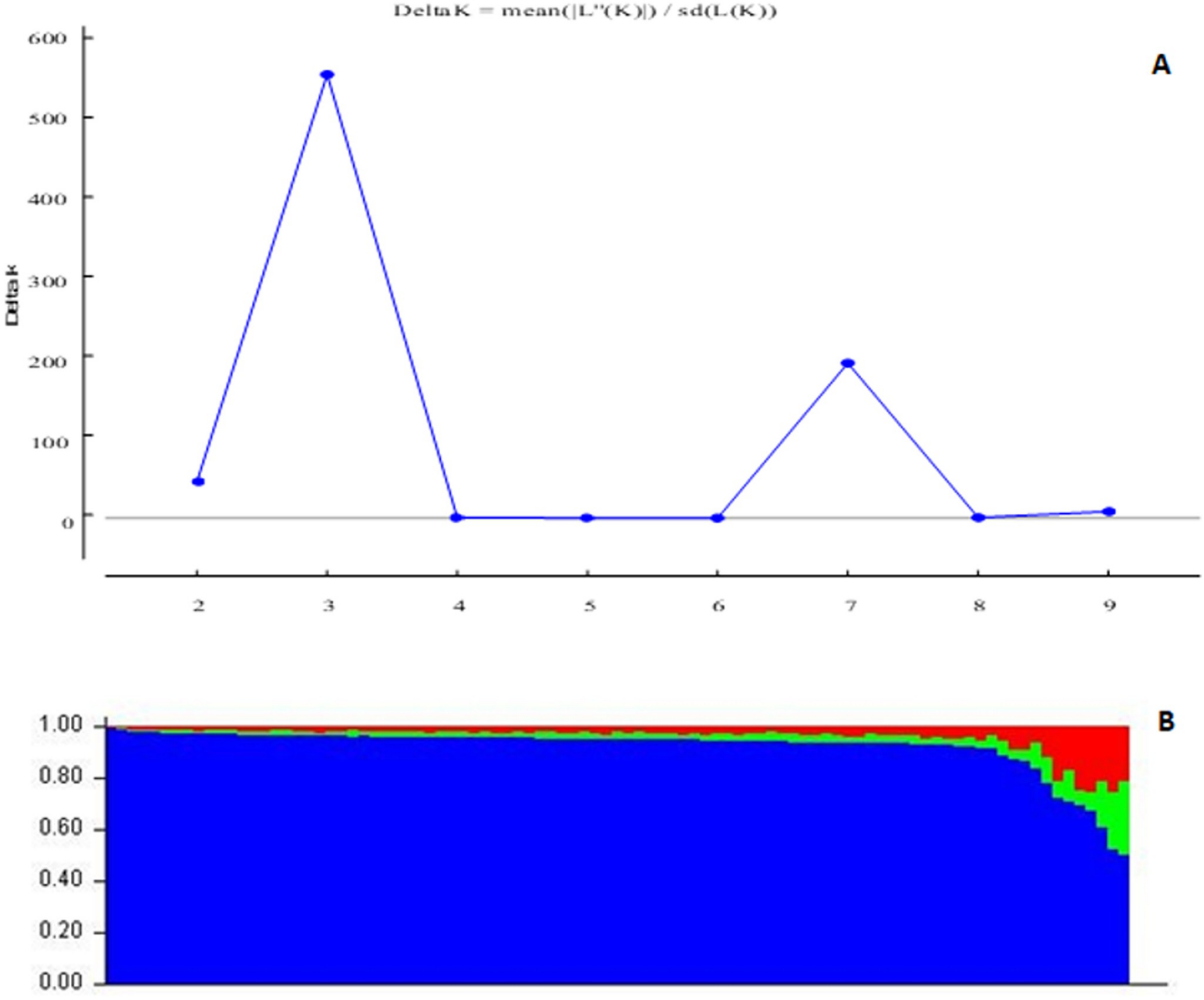
Population inference among the 81 pigeonpea genotypes based on 4122 SNP markers showing (A) likelihood and delta K values for the number of assumed clusters (B) population structure at K = 3.

The SNPs resoved, reolved three distinct sub-populations among the 81 accessions (Fig 1A and 1B) based on the highest ΔK value at K = 3 following the Evanno method. Sub-population 1 consisted of 15% of genotypes and comprised breeding lines. Sub-population 2 had 5% of the genotypes, mainly cultivars, while sub-population 3 consisted of mainly landraces.

The genetic differentiation among the populations ranged from -0.011 to 0.002 (Table 3). The highest genetic differentiation (Fst) was observed between sub-population 1 (breeding lines) and sub-population 2 (cultivars). In contrast, the lowest Fst value was observed between sub-population 2 (cultivars) and sub-population 3 (landraces). The analysis of molecular variance (AMOVA) (Table 4) among 81 pigeonpea genotypes indicated that 2.7% of the variation was due to genetic differences among the sub-populations, while 97.3% of the variation was due to the genetic differentiation among individuals within the sub-populations.

**Table 3:** Population genetic differentiation/distance (Fst) for 81 pigeopea genotypes.

Population	G1	G2	G3
**G1**	-		
**G2**	0.002	-	
**G3**	-0.011	-0.014	-

G1 = breeding lines, G2 = cultivars, G3 = breeding landraces

**Table 4:** Analysis of molecular variance among three populations in 81 pigeonpea genotypes.

Source of variation	DF	SS	MS	Estimated variance	Variance (%)	P-value
Among populations	2	229.25	114.63	0.34	2.70	0.300
Within populations	78	8377.19	107.40	107.40	97.30	0.003
**Total**	**80**	**8606.44**		**107.74**	**100**	

DF = degrees of freedom, SS = sum of squares, MS = mean square, P-value = significance level

The results obtained from genetic structure analysis based on the phylogenetic tree resolved the 81 genotypes into three groups (Fig 1). Group III was composed of a large number (45) of genotypes, followed by Group I (31) and Group II, which had the least (5) genotypes. Genotype grouping represented a mixture of landraces, breeding lines, and cultivars. However, the genotypes in Group I mainly were characterized by early maturity, while Groups II and III were composed of medium and late maturing genotypes, respectively.

### Phenotyping

Genotypic variation was significant for most quantitative traits (Table 5). The earliest flowering genotypes flowered in 48 days, while the latest genotype took 195 days to flower. On average, the DTF was 111 days. Similarly, there was a wide variation in days to maturity (DTM), which exhibited a 15.64% coefficient of variation. The genotypes included short and tall plants. The shortest genotype was 0.73 m tall compared to the tallest genotype, which reached 3 m. The yield-related traits such as number of pods per plant, number of racemes per plant, number of secondary branches, and 100 seed weight also exhibited high coefficients of variation, showing their wide variability among the genotypes. The mean grain yield was 1.14 t ha^-1^, ranging between 0.11 t ha^-1^ and 3.67 t ha^-1^.

**Table 5:** Summary statistics for qualitative and quantitative traits of 81 pigeonpea genotypes evaluated in six environments in Malawi.

	DTF	PH	DTM	NPB	NPP	NRP	NSB	NSP	PDL	HSWT	GYD	FMC	FSP	GH	PC	SCP	SEC	SMC	SS
MEAN	110.95	167.51	155.67	14.52	94.19	148.98	6.83	5.4	8.1	14.68	1.14	2.94	0.89	2.15	2.17	1.79	2.52	1.87	2.21
MEDIAN	112.5	167	157	14	87	113.5	5.5	5	8	15	1.12	3	0	2	2	1	2	2	3
MIN	48	73	0	0	0	32	0	0	0	0	0.11	0	0	0	0	0	0	0	0
MAX	195	299	262	47	400	740	56	8	12	29	3.67	6	4	3	5	5	10	9	3
RANGE	147	226	262	47	400	708	56	8	12	29	3.57	6	4	3	5	5	10	9	3
Q1	102	142	143	12	59	74	3	5	8	12	0.67	3	0	2	1	1	2	2	1
Q3	124	194.65	168	17	117.5	179	9	6	9	18	1.5	3	2	3	3	3	2	2	3
SD	20.03	37.52	24.34	4.24	52.32	112.57	6.01	0.98	1.51	4.41	0.59	1.03	1.22	0.6	1.26	1.06	1.49	0.79	0.92
SEM	0.64	1.2	0.78	0.14	1.68	3.61	0.19	0.03	0.05	0.14	0.02	0.03	0.04	0.02	0.04	0.03	0.05	0.03	0.03
VAR	401.39	1407.56	592.52	17.96	2737.32	12672.11	36.1	0.97	2.29	19.43	0.35	1.06	1.49	0.36	1.58	1.13	2.23	0.63	0.84
%CV	18.06	22.4	15.64	29.19	55.55	75.56	87.95	18.22	18.7	30.02	51.74	35.1	137.08	28	58.04	59.13	59.28	42.29	41.47
SKEW	-0.53	0.02	-0.53	1.32	1.52	2.04	2.32	-1.42	-2.19	-0.72	0.64	-0.21	0.93	-0.1	0.43	0.82	2.57	2.53	-0.54
KURTOSIS	0.99	-0.24	3.26	6.96	4.48	4.65	10.14	6.85	10.52	1.49	0.25	0.35	-0.65	-0.25	-1.07	-0.33	6.77	15.27	-1.32

DTF = days to 50% flowering, DTM = days to 75% maturity, PH = plant height in centimetres, DTM = days to maturity, NPB = number of primary branches, NSB = number of secondary branches per plant, NRP = number of racemes per plant, NPP = number of pods per plant, PDL = pod lenghth, NSP = number of seeds per pod, GYD = grain yield, HSWT = 100 seed,FMC = flower main color, FSP = flower streak pattern, GH = growth habit, PC = pod color, SCP = seed color pattern, SEC = seed eye color, SS = seed shape, Min = minimum, Max = maximum, Q1 = quartile one, Q3 = quartile three, SD = standard deviation, SEM = standard error of the mean, VAR = variation, CV = coefficient of variation

Using morphological attributes, the phenotypic diversity assessment [24] grouped the genotypes into three distinct clusters (Fig 3). Cluster 2 recorded the highest number (51) of genotypes, followed by Cluster 1 [37] and Cluster 3 (3). The genotypes in Cluster 1 included two landraces from Malawi; MWPLR 14 (G41) and MWPLR 24 (G26), and one collection from Tanzania, TZA 197 (G76), both with medium maturity. The genotypes in Clusters 1 and 2 were a mixture of landraces, breeding lines, and cultivars. However, genotypes in Cluster 2 were mainly medium to late maturing, which included Babati (G49), Hombolo (G78), Sauma (G81), TZA 5557 (G50), ICEAP 0673/1 (G1), MZ2/9 (G23), among others. Cluster 1 had most of the early maturing genotypes such as ICEAP 87105 (G42), ICEAP 01170 (G6), ICEAP 87091 (G58), ICEAP 01150 (G22), ICEAP 00612 (G64), ICEAP 01172/1 (G24), ICEAP 01146/01 (G18).

#### Combined analysis of phenotypic and genotypic data

The phylogenetic tree generated from the phenotypic data was compared to the genotype grouping based on the SNP data (S2 Fig). The results show that only 13.5% of the accessions maintained the same position across the hierarchical clusters. There was a clear indication of the grouping patterns and membership delineated by the phenotypic and genotypic datasets. Thirty-seven genotypes representing 45.7% of the genotypes maintained their groups across the phenotypic and genotypic hierarchical clusters. Using the combined phenotypic and molecular data, genetic diversity assessment clustered the accessions into three groups (Fig 4). Groups I, II, and III comprised of 34, 7, and 40 genotypes, in that order. The genetic grouping represented a mixture of landraces, breeding lines, and cultivars.

## Discussion

Preliminary evaluation of the pigeonpea germplasm revealed significant genetic variation based on phenotypic traits [18]. Such variation is essential but subject to influence by environmental conditions that confound selection. Morphological and agronomic traits are essential in germplasm preliminary description and classification for plant breeding programs [12]. Onwobiko [38] reported that both qualitative and quantitative characters could be used to establish the morphological variations in cowpea germplasm. A follow-up assessment using molecular markers is necessary to confirm the observed phenotypic divergence and grouping underlying genetic basis.

Determination of genetic diversity among genotypes, populations, and gene pools is essential to identify unique individuals as sources of genes for improving quantitative or qualitative traits. Several studies have been conducted that assessed the genetic diversity in pigeonpea using morphological descriptors [24, 39, 40], biochemical markers [12, 22, 41], and DNA-based molecular markers [8]. This study used SNP markers to elucidate genetic diversity and grouping using expected heterozygosity and the polymorphic information content (PIC). These parameters measure alternate allele representation and different allele combinations among genotypes in a breeding population [42]. The PIC values indicate the allelic diversity within individuals and the usefulness of markers for tracking between offspring and parental genotypes. The gene diversity for the haploid markers estimates the average genetic distance among individuals in the population [43]. In the present study, the PIC values ranged from 0.00 to 0.38 (Table 2), showing that the germplasm displayed various levels of allelic diversity. However, the observed average PIC value of 0.11 indicates that the overall diversity was moderate. The average PIC value observed in this study was comparable to what was previously reported in 184 pigeonpea germplasm obtained from the ICRISAT genebank [14].

Similarly, relatively low PIC values were reported in common bean and cowpea germplasms, respectively in Malawi and Zambia [44, 45] due to the low genetic polymorphism detected in the assessed accessions. The low PIC values obtained using SNP markers could be due to their bi-allelic nature which restricts the range of PIC values between 0 and 0.50 only [44]. Conversely, Yang et al. [22] reported high informative DArT markers with PIC values ranging from 0.002–0.50, for 232 pigeonpea accessions, including cultivated and wild species, respectively. The relatively high PIC values suggested that the sampled DArT markers were of good quality and hence can be effectively used in molecular systematics and biodiversity studies.

The negative inbreeding coefficient values indicate the presence of considerable heterozygosity in the test populations owing to the inherent outbreeding of pigeonpea. The excess heterozygosity observed in this study may have resulted from the markers deviating from the expected Mendelian ratios, referred to as “segregation disorders”. Although the actual cause of this disorder is unknown, sampling error favouring heterozygotes is among the suspected causes of excessive heterozygosity [37, 46]. However, this is not unusual, including in recombinant inbred lines of autogamous crop species. Pigeonpea has a varied rate of out-crossing (5–70%) depending on genotype, insect activities, weather conditions and their interactions [47]. This promotes inter- and intra-species crossing and the occurrence of segregation disorders [37]. Low heterozygosity values of 0.27 were reported among pigeonpea genotypes evaluated in Tanzania [48]. Conversely, the observed heterozygosity value of 0.22, GD (0.14), and MAF (0.12) (Table 2) suggested that some of the test lines were comparatively homozygous. The complete flower system of the crop renders autogamous mating system and some degree of self-pollination and homozygosity.

The presence of low levels of heterozygosity among individuals and the low frequency of rare variants in the population could present bottlenecks for breeding. Adequate genetic diversity facilitates the adaptation of populations to changes in environmental conditions [49]. High heterozygosity and rare variants provide opportunities for optimal gene recombinations during cultivar development [50]. The AMOVA revealed that much (97.3%) of the genetic variation was among individuals within the populations (Table 4), which shows that individual selection of superior genotypes as parental lines for developing breeding populations would be more effective. The low genetic divergence (2.7%) among the three populations could result from selective breeding that has increased similarity among different genotype groups such as breeding lines, elite lines, and released varieties due to common parentage. Other studies have found higher genetic variation among populations that could facilitate inter-cluster crossing during breeding population development. For instance, Obua et al. [40] found that genetic variation among soybean populations accounted for 54% of the total genetic variation. Similarly, 51% of the genetic variation was attributed to the difference among populations of a panel of common bean that consisted mostly of landraces [38]. The discrepancies in the results reported by different authors could be attributed to differences in sample sizes and origins of accessions of the same species. Nevertheless, the population used in this study exhibited moderate diversity, heterozygosity, and PIC, which could be bottlenecks for pigeonpea improvement.

**Fig 2:**
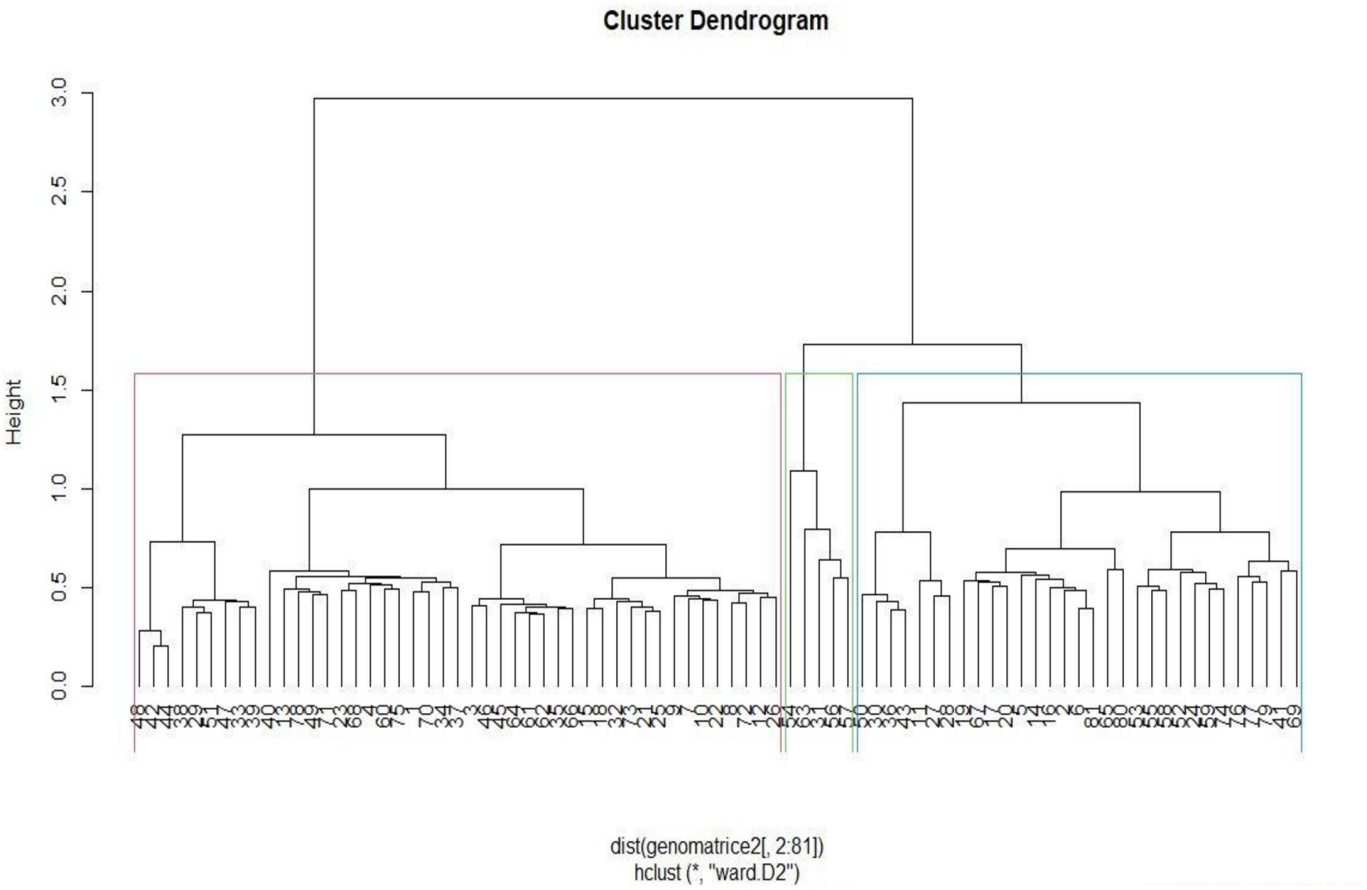
Hierarchical cluster dendrogram showing the genetic relationships among 81 pigeonpea accessions using 4122 SNP markers. See Table 1 for code of genotypes.

The highest delta value occurred at K = 3 in the population structure analysis (Fig 1), showing that the 81-pigeonpea genotypes could be delineated into three sub-populations. The delineation of the genotypes was irrespective of the sources of collection, which indicated that gene flow had transcended geographical boundaries due to the frequent exchange of genetic resources spearheaded by international genebanks. Similarly, the dendrogram (Fig 2) grouped the genotypes into three sub-populations with no distinction among breeding lines, landraces, and cultivars. Selective breeding using elite lines from a narrow genetic base has increased similarity among cultivars and breeding lines. It is relatively easier to breed new cultivars using elite lines than landraces or wild relatives containing undesirable traits that could take continuous selection cycles. This causes a lack of divergence among different genotypes and genetic erosion for important traits. The lack of distinct grouping among test genotypes showed that there were possibly admixtures in the groups that resulted in low genetic differentiation (Fst) between the groups (Table 3). The Fst value obtained in this study was lower than 0.15, which is considered a reasonable lower threshold for genetic differentiation in pigeonpea [51]. The low Fst value shows that the clusters are not genetically divergent, and crosses should be designed based on individual phenotype and genotype data rather than inter-cluster mating.

**Fig 3:**
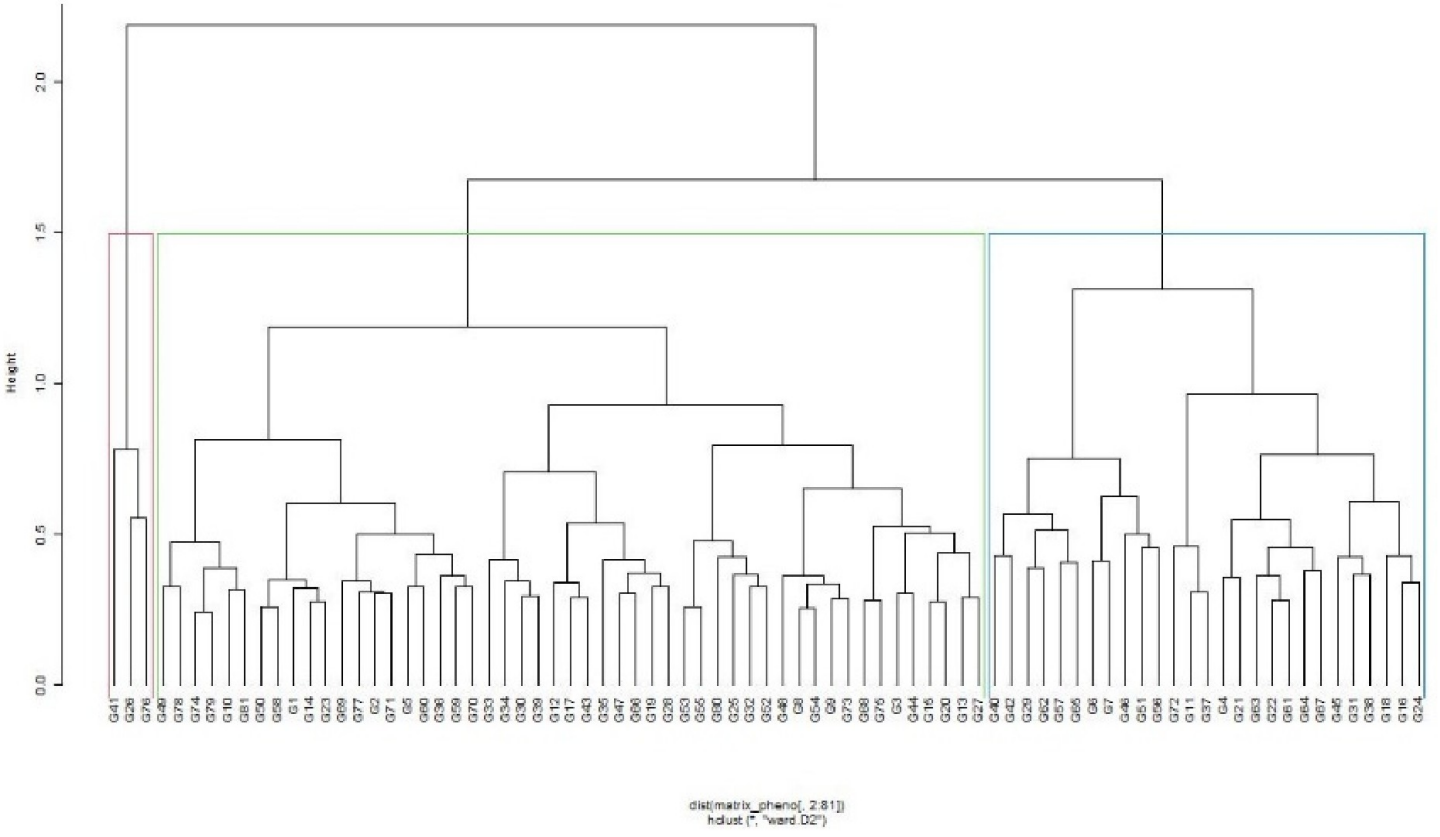
Hierarchical cluster dendrogram showing genetic grouping among 81 pigeonpea genotypes evaluated in six environments in Malawi based on phenotypic traits. See Table 1 for code of genotypes.

The grouping of genotypes into three clusters (Figs 2 and 3) using SNP markers and morphological traits revealed a mixture of breeding lines, landraces, and cultivars in each group. This could be attributed to the geographical proximity between the two countries, Malawi and Tanzania, where the landraces were collected. Farmers between the two countries have a long history of sharing germplasm. In addition, the breeding lines from ICRISAT were developed using some parents selected from the landraces from Tanzania and Malawi hence, the genotypes in the germplasm were likely to be related. In a related study [52], reported high similarity between the cultivars due to direct selection or selections from the crosses involving germplasm lines from ICRISAT. Similarly, Yang et al. [22] reported little variation among the cultivated pigeonpea collected in Africa and Khurshid et al. [53] reported significant genetic variation among the 30 Pakistan oilseeds *Brassica* cultivars based on PCR-based DNA polymorphism. The tested cultivars were clustered into two major groups and four sub-groups. However, there was a narrow genetic base among the Pakistan oilseeds *Brassica* cultivars.

**Fig 4:**
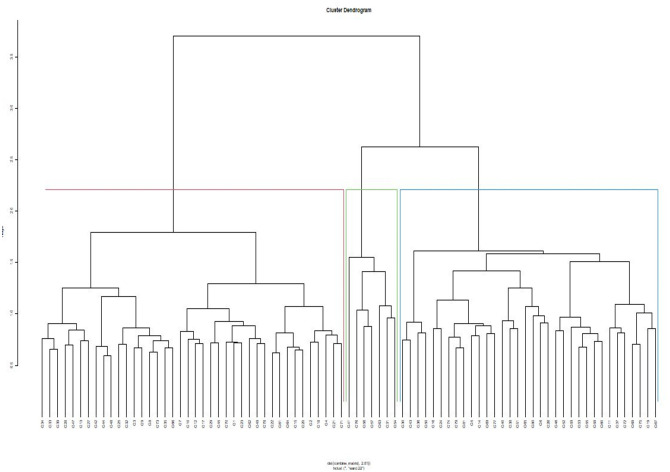
Hierarchical cluster based on the combined phenotypic and molecular data in 81 pigeonpea genotypes. See Table 1 for the code of genotypes.

A joint analysis of phenotypic and genotypic data was conducted to capture the genetic variability and grouping of the test population. The comparison between the phenotypic and genotypic information showed that 45.7% (Fig 4) of the accessions evaluated maintained their membership across the phenotypic and molecular clustering, showing that the phenotypic and molecular matrices differed but were complementary. The use of both derived clusters would increase precision in selecting divergent parents, from the groups for breeding. Increased precision in selection using a combination of genotypic and phenotypic data has been previously reported in legumes such as cowpea [45]. New breeding populations can be developed by hybridization among the three divergent genetic groups, especially those that have maintained their groups, to broaden the genetic base as part of a pigeonpea pre-breeding program in Malawi.

## Conclusions

The present study assessed the genetic diversity and grouping among the 81-pigeonpea accessions sourced from Malawi, Tanzania, and ICRISAT/Kenya. The genetic diversity and grouping of the test populations were confirmed using morphological traits, SNPs data, and joint analysis. The test genotypes were grouped into three genetic clusters, enabling the selection of divergent parents for hybridization and the development of new pigeonpea breeding populations in Malawi. There is a need to create a new genetic variation or introgress genes from genetically contrasting parents to harness the genetic variation in the presently assessed pigeonpea population.

## Supporting information

S1 Fig Diversity analysis data for each chromosome and whole genome data. (DOCX)

S2 Fig Comparison of hierarchical cluster dendrograms based on phenotypic traits (A) and SNPs data (B) in 81 pigeonpea genotypes. See Table 1 for the code of genotypes.
(DOCX)

S1 Table Descriptors for the pigeonpea qualitative and quantitative traits. (DOCX)

S1 File Phenotypic data. (XLSX)

S2 File Copy of metadata. (XLSX)

S3 File Metadata. (TXT)

S4 File Report_DPp18-2627_SilicoDArT_1. (CSV)

S5 File Report_DPp18-2627-SNP_2. (CSV)

S6 File Report_DPp18-2627_SNP_singlerow_2. (CSV)
