# Supplementary figures and images for: Genetic diversity and grouping of pigeonpea [Cajanus cajan Millspaugh] Germplasm using SNP markers and agronomic traits
Source: PLoS One. 2022 Nov 3;17(11):e0275060. doi: 10.1371/journal.pone.0275060 (PMC9632774; doi:10.1371/journal.pone.0275060)

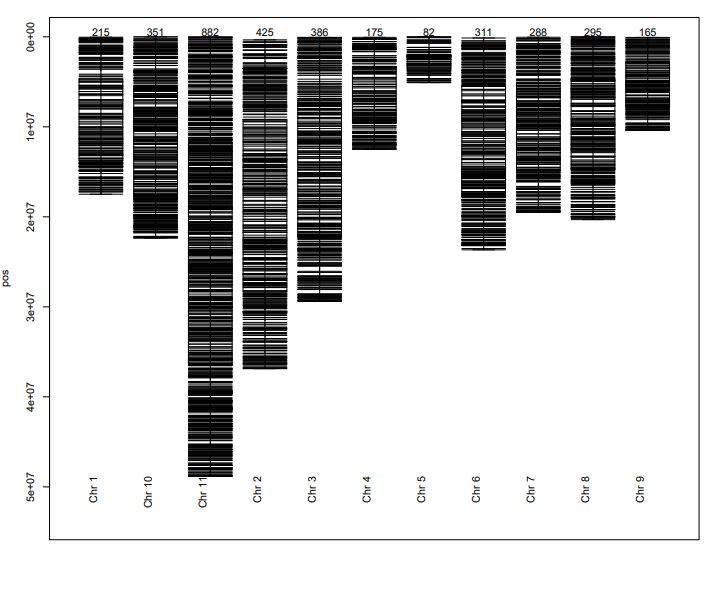


**S1 Fig. Diversity analysis data for each chromosome and whole genome data**

Supplement: S1 Fig — (DOCX) [file pone.0275060.s001.docx]
