# Supplementary material for: Genetic diversity and grouping of pigeonpea [Cajanus cajan Millspaugh] Germplasm using SNP markers and agronomic traits
Source: PLoS One. 2022 Nov 3;17(11):e0275060. doi: 10.1371/journal.pone.0275060 (PMC9632774; doi:10.1371/journal.pone.0275060)
Supplement: S2 Fig — See Table 1 for the code of genotypes. (DOCX) [file pone.0275060.s002.docx]

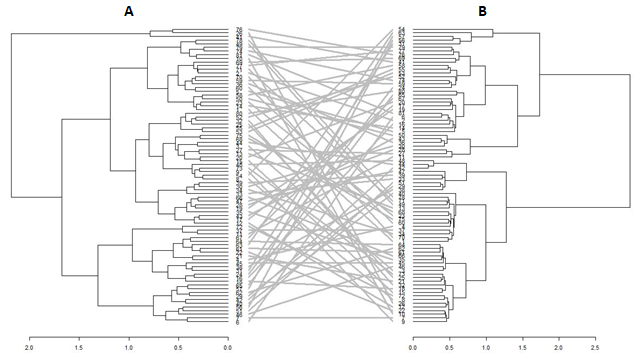


**S2 Fig. Comparison of hierarchical cluster dendrograms based on phenotypic traits (A) and SNPs data (B) in 81 pigeonpea genotypes.**

See Table 1 for the code of genotypes
