# Supplementary material for: Genetic diversity and grouping of pigeonpea [Cajanus cajan Millspaugh] Germplasm using SNP markers and agronomic traits
Source: PLoS One. 2022 Nov 3;17(11):e0275060. doi: 10.1371/journal.pone.0275060 (PMC9632774; doi:10.1371/journal.pone.0275060)
Supplement: S1 Table — (DOCX) [file pone.0275060.s003.docx]

**S1 Table. Descriptors for the pigeonpea qualitative and quantitative traits**

| **Traits** | **Code** | **Description** |
| --- | --- | --- |
| **Qualitative traits** | | |
| Growth habit | GH | 1=Compact (erect), 2=semi-spreading (semi-erect) or 3=spreading |
| Flower streak pattern | FSP | 0= no streaks, 1=Sparse, 2=medium and 3=dense streaks, 4= uniform coverage of second color |
| Flower base/main colour | FMC | 1=Ivory (green white), 2= light yellow, 3= yellow, 4= orange, 5= red, 6= purple |
| Pod colour | PC | 1=Green, 2=purple, 3=mixed (green +purple) and 4=dark purple |
| Seed colour pattern | SCP | 1= Plain, 2= mottled, 3=speckled, 4=Mottled and speckled, 5=ringed |
| Seed main colour | SMC | 1= white (yellow white), 2= cream (grey white), 3= orange, 4=brown, 5=grey, 6= purple, 7= black |
| Seed eye colour | SEC | 1= Purple, 2= light brown, 3= reddish brown, 4= grey/dark, 5= cream/white |
| Seed shape | SS | 1=Oval, 2=pea-shape, 3= square/angular, 4= elongate |
| **Quantitative traits** |  |  |
| Plant height | PH | Measured in cm from plant base to the tip of the main stem |
| Days to 50% flowering | DTF | Number of days from sowing until when 50% of the plants have at least one open flower |
| Primary branches | NPB | Average number of primary branches of 10 randomly selected and tagged plants |
| Secondary branches | NSB | Average number of secondary branches of 10 randomly selected and tagged plants |
| Days to 75% maturity | DTM | Number of days from sowing until when 75% of the pods in a plot turn brown |
| Number of seeds per pod | NSP | Average number of pods per plant from 10 randomly selected and tagged pods |
| Number of pods per plant | NPP | Average number of pods from 10 randomly selected and tagged plants |
| Number of racemes per plant | NRP | Average number of racemes from 10 randomly selected and tagged plants |
| Grain yield (t/ha) | GYD | Weight of the grain harvested in a plot extrapolated to t/ha |
| 100 seed weight (g) | HSWT | Weight of a random sample of 100 grain |
